# A systematic review of the psychological and social benefits of participation in sport for children and adolescents: informing development of a conceptual model of health through sport

**DOI:** 10.1186/1479-5868-10-98

**Published:** 2013-08-15

**Authors:** Rochelle M Eime, Janet A Young, Jack T Harvey, Melanie J Charity, Warren R Payne

**Affiliations:** 1 Institute of Sport, Exercise and Active Living, Victoria University, PO Box 14428, Melbourne, Victoria 8001, Australia; 2 School of Health Sciences, University of Ballarat, PO Box 663, Ballarat, Victoria 3353, Australia

**Keywords:** Sport, Health, Psychological, Psychosocial, Social

## Abstract

**Background:**

There are specific guidelines regarding the level of physical activity (PA) required to provide health benefits. However, the research underpinning these PA guidelines does not address the element of social health. Furthermore, there is insufficient evidence about the levels or types of PA associated specifically with psychological health. This paper first presents the results of a systematic review of the psychological and social health benefits of participation in sport by children and adolescents. Secondly, the information arising from the systematic review has been used to develop a conceptual model.

**Methods:**

A systematic review of 14 electronic databases was conducted in June 2012, and studies published since 1990 were considered for inclusion. Studies that addressed mental and/or social health benefits from participation in sport were included.

**Results:**

A total of 3668 publications were initially identified, of which 30 met the selection criteria. There were many different psychological and social health benefits reported, with the most commonly being improved self-esteem, social interaction followed by fewer depressive symptoms. Sport may be associated with improved psychosocial health above and beyond improvements attributable to participation in PA. Specifically, team sport seems to be associated with improved health outcomes compared to individual activities, due to the social nature of the participation. A conceptual model, Health through Sport, is proposed. The model depicts the relationship between psychological, psychosocial and social health domains, and their positive associations with sport participation, as reported in the literature. However, it is acknowledged that the capacity to determine the existence and direction of causal links between participation and health is limited by the fact that the majority of studies identified (n=21) were cross-sectional.

**Conclusion:**

It is recommended that community sport participation is advocated as a form of leisure time PA for children and adolescents, in an effort to not only improve physical health in relation to such matters as the obesity crisis, but also to enhance psychological and social health outcomes. It is also recommended that the causal link between participation in sport and psychosocial health be further investigated and the conceptual model of Health through Sport tested.

## Background

Regular participation in physical activity (PA) is imperative for good health. Active people benefit from higher levels of health-related fitness and are at lower risk of developing many different disabling medical conditions than inactive people [1,2]. It is widely acknowledged that the health benefits of participation in PA are not limited to physical health but also incorporate mental components [1,2].

Extensive research has resulted in clear recommendations of the level of PA required to produce health benefits [1,3]. There are specific health-related recommendations for children and adolescents distinct from those for adults. For people aged 5–17 years it is recommended that they undertake moderate or vigorous activities for at least 60 minutes per day [4]. Regular maintenance of this level of activity by children and adolescents can result in increased physical fitness, reduced body fat, favourable cardiovascular and metabolic disease risk profiles, enhanced bone health and reduced symptoms of depression and anxiety [1]. Whilst many different health benefits of participation in PA are acknowledged, the vast majority of research has focused on the physical health benefits of participation in PA, with less research focused on the mental and social health aspects. Although mental health benefits have been referenced in recent guidelines, to date ”insufficient evidence precludes conclusions about the minimal or optimal types or amounts of physical activity for mental health” [1] (Part G Section 8 p39).

Even though the World Health Organisation definition of health (2006) incorporates physical, mental and social health domains, the research providing evidence to the PA guidelines does not specifically address social health. However, the literature informing PA guidelines does suggest that aspects such as social support may contribute to some of the explanations of mental health outcomes [1].

Leisure-time PA is one domain of PA. Sport is one type of leisure-time PA which is organised and usually competitive and played in a team or as an individual [5]. Participation in sport is very popular among children. However there is evidence that participation in sport peaks at around 11–13 years before declining through adolescence [6,7]. Conversely, there is research indicating that children who are active through sport are more likely to be physically active in adulthood than those who do not participate in childhood sport [8,9]. Further, substantial public investment in sport development has been justified in terms of a range of health benefits [10], but without a clear understanding of the best way to achieve maximum health benefits - both mental and physical.

Extensive research has been conducted on the determinants of participation in PA [6,11] and on interventions that attempt to increase PA participation [12], with relatively little research focusing more specifically on sport [9,13]. Also, with regard to the health benefits of PA, the research has generally not extended to the mental and social health benefits of sport participation in particular.

A conceptual model in the public health area has been defined as “diagram of proposed causal linkages among a set of concepts believed to be related to a specific public health problem” [14] (p163). Determinants of PA are increasingly being understood using socio-ecological models, whereby intrapersonal, interpersonal, organisational, environmental and policy variables are identified as influences on participation [15-18]. As Earp and Ennett (1991) explain, conceptual models in health do take an ecological perspective, implying that behaviours or health outcomes result from the interaction of both individual and environmental determinants [14,19]. In terms of the sport and health nexus, we are not aware of a conceptual model that depicts the specific mental and social health outcomes of sport participation. Conceptual models have been developed which show the relationship between different types of PA, including sport, and the intensity and context of participation [20], however they do not extend to the health benefits of participation. In one systematic review of the effectiveness of interventions to increase physical activity, a conceptual model of the relationship between interventions, modifiable determinants, immediate outcomes and health outcomes was developed [21]. However, this study did not specifically identify sport. Furthermore, there are many clinical conceptual models depicting health outcomes of clinical conditions, however they do not focus on the general population or on preventive health or health promotion [22].

Firstly, this paper presents the results of a systematic review investigating the psychological and social benefits of participation in sport for children and adolescents. Secondly, the information obtained in the systematic review has been used to develop a conceptual model: the conceptual model of Health through Sport, for children and adolescents.

## Methods

The criteria for considering studies for this review were as follows.

Inclusion criteria were:

1. Studies published in English between Jan 1990 and May 2012 inclusive.

2. Original research or reports published in peer review journals or government or other organisational publications which reported primary data.

3. Studies that presented data that addressed mental and/or social health benefits from participation in sport. In this context, the following definitions were adopted: ‘sport’ - “a human activity of achieving a result requiring physical exertion and/or physical skill which, by its nature and organisation, is competitive and is generally accepted as being a sport” [23]. ‘health’ – “a state of complete physical, mental and social well-being and not merely the absence of disease and infirmity” [24]; ‘mental’ - “of or referring to the mind or to the processes of the mind, such as thinking, feeling, sensing, and the like” [25] (p475) ‘mental health’ – "Mental Health refers to a broad array of activities directly or indirectly related to the mental well-being component included in the WHO's definition of health…It is related to the promotion of well-being, the prevention of mental disorders, and the treatment and rehabilitation of people affected by mental disorders” [26,27] ‘social’: “Relating to the interactions of individuals, particularly as members of a group or a community ” [25] (p475); ‘social health’: “That dimension of an individual’s well-being that concerns how he gets along with other people, how other people react to him, and how he interacts with social institutions and societal mores.” [28] (p 152). In this study, we also used the following terms: ‘psychological’ – as a synonym for ‘mental’; and ‘psychosocial’ - “…any situation in which both psychological and social factors are assumed to play a role” [29] (p638).

4. Studies where the data pertained to the individual level (i.e. for persons versus communal or national level).

Exclusion criteria were:

1. Studies or reports that addressed ‘exercise’ , ‘physical activity’ , ‘physical education’ , or ‘recreation’ , and not sport. Definitions of these terms are: ‘Exercise’ –“physical activity that is planned, structured, repetitive, and purposive in the sense that improvement or maintenance of one or more components of physical fitness is an objective” [27] (p128); ‘Physical activity’ - “bodily movement produced by skeletal muscles that results in energy expenditure” [27] (p126); ‘Physical education’ - “a sequential, developmentally appropriate educational experience that engages students in learning and understanding movement activities that are personally and socially meaningful, with the goal of promoting healthy living” [30] (p8); ‘Recreation’ – “pleasurable activity” [31] (p. 915).

2. Research/reports that addressed participation in ‘adapted’ sports (i.e. sport participation for persons with a physical and/or intellectual disability, such as wheelchair tennis).

3. Research/reports that addressed sub-populations subject to specific risks (i.e. studies with heroin users, ‘at risk’ individuals etc.).

4. Research/reports that addressed rehabilitation from, or management of, injury or illness.

5. Research/reports that addressed spectators, coaches or sports administrators.

6. Research/reports that addressed elite sports participants

7. Research/reports that addressed ‘sport development’ programs that have an educational objective.

8. Book chapters, abstracts, dissertations and conference proceedings.

### Search methods for identification of studies, reports and publications

A systematic search of 14 electronic databases (AUSPORT, AusportMed, CINAHL, Cochrane Library, EBSCHOHost Research Databases, Health Collection, Informit, Medline Fulltext, PsycARTICLES, Psychology and Behavioral Sciences Collection, PsycINFO, PubMed, Scopus, SPORTDiscus Fulltext) was conducted in June 2012. We also consulted with the Australian Sports Commission to search the National Sports Information Centre records in order to identify relevant reports, publications and research not located through the search of the electronic databases cited above. Further, we conducted an internet search using the Google Scholar search engine (http://www.googlescholar.com) to locate studies in the Medicine, Social Sciences, Arts and Humanities subject areas. The Google Scholar search engine was also used to search for recognised International, National and State reports and publications that directly addressed the topic under consideration.

To search the electronic databases a combination of keywords and search terms was adopted. These key words and search terms were formulated by the authors of this systematic review as those they considered directly addressed the topic under consideration. These keywords and search terms constituted four groups, namely:

Group 1: sport

Group 2: health

Group 3: value, benefit, effect, outcome

Group 4: psychology, depression, stress, anxiety, happiness, mood, quality of life, social health, social relations, well, social connect, social functioning, life satisfaction, mental health, sociology, social.

Accordingly where possible, the database searches consisted of key words from Group 1 AND Group 2 AND Group 3 AND Group 4. The truncation symbol was added to the most basic word stem for each keyword to ensure all associated terms were included in the search.

### Study selection

**Figure 1:**
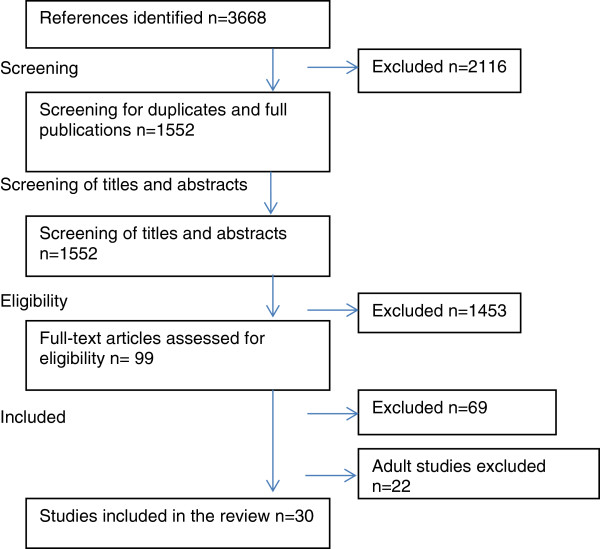
Stages of study selection.

Figure 1 provides a summary of the stages of study selection. Titles and abstracts of potentially relevant articles were screened by JY. Authors, JY and RE examined all full-text articles, and assessed the studies to ensure that they met the inclusion criteria. Any discrepancies were resolved through discussion between the two reviewers. Consensus was obtained for all included articles. After reviewing the selected studies it was decided, given the breadth and complexity of the research domain, that studies focusing on children and adolescents should be reviewed separately from studies focusing on adults, This review focuses on children and adolescents only; studies that stated that they specifically investigated children and/or adolescents, but not adults (18 or above), were included.

### Data collection and analysis

Data extracted from each of the studies included: study design and methodology; sample size; country of origin; age of participants; cohort of participants; gender of participants; study aim; sport variable; other PA variables; theoretical construct; key findings in relation to psychological and social health outcomes.

### Assessment of study quality

Study quality was objectively appraised using the Downs and Black checklist [32]. This checklist has been used in other systematic reviews within the physical activity and health field [33,34]. This checklist includes 27 items grouped into categories: reporting (10), external validity (3), internal validity - bias (7), internal validity – confounding (6), and power (1). Twenty five items are scored as 1 (compliance) or 0 (non-compliance or inability to determine compliance); one item about confounding is scored as 2 (full compliance), 1 (partial compliance) or 0 (non-compliance or inability to determine compliance); and the item concerning power is scored (via a more complex algorithm) on a scale of 0–5.

Because most of the studies we reviewed did not involve interventions, a number of the items on the Downs and Black checklist were not generally applicable. We substituted a simpler power item (presence or absence of reference to a power analysis), and scored all items as 0, 1 or NA (not applicable). We calculated a summary quality score for each paper (except the two qualitative papers for which only five items were applicable) by expressing the number of compliant items as a percentage of the number of applicable items. We included these scores (ranging from 33% to 88%) in Table 1, and used the insights we gained through the scoring process in our discussion of study quality.

**Table 1:** Studies investigating the psychological and social health benefits of participation in sport for children and/or adolescents

**Ref. & Year**	Design*	**Method**	**Sample (n)**	**Country**	**Age (yrs)**	Cohort**	Sex***	**Aim**	**Sport**	**Other PA**	**Theory**	**Key finding(s)**	**Psychological, social health outcomes**	**Score (%)**
[35] 2011	Quant.	Long.	739	USA	11-15 & 15-18	Adol.	B	Explore associations between sport & suicide ideation & attempts	Sport	No sport	-	Youth involved in sport in both middle & high school had lower odds of suicidal ideation than non-sport participants	Fewer suicide attempts	78
[36] 2011	Qual.	Cross.	17 parents & 18 Children	Canada	Child. M 13 adults M 45	Child. & adult	B	Investigate perceptions of benefits of youth sport participation & challenges with providing children with sporting opportunities	Sport	-	Ecological approach, Positive Youth Development	Parents & children reported sport participation associated with a range of personal & social developmental benefits including emotional control & confidence & making new friends, relationships & social skills	Social benefits (relationship with coaches, friends, teamwork/social skills), Personal benefits (emotional control, exploration, confidence)	NA
[37] 2011	Quant.	Long.	208	Swiss	7-8 & parents	Child. & parent	B	Investigate role of sport as mediating onset or development of social anxiety	Extracurricular sport	Individual or no sport	Antonovsky’s (1997) Salutogenesis model & Bandura’s (1977)Social Learning theory	Children in team sports reported a reduction in social anxiety	Reduced anxiety	78
[38] 2010	Quant.	Cross.	325	USA	-	Adol.	B	Compare health-related quality of life between athletes (school or club sports) & non-athletes	School or club sport	Non-school or club sport	-	Athletes (school or club sports) reported higher social functioning, mental health & happiness compared with non-athletes	Mental health	53
[39] 2010	Quant.	Cross.	31,117	USA	6-11	Child.'s parent	B	Investigate association between participation in out-of-school activities & behaviour	Sport team/lesson & sport club/organisation	No outside school activity	-	Children who participated in sports & clubs had greater social competence during middle childhood compared with children who did not participate in any sports or clubs outside of school activities	Social skills, problem behaviour overall, try to resolve conflicts, show respect for teachers & neighbours	87
[40] 2009	Quant.	Long.	1357	USA	M 11 Wave 1	Adol.	B	Assess relations among sports participation, other extracurricular activities & indicators of youth development	Sport	Other extracurricular activities	Theory of Positive Youth Development	Participation in a combination of youth & youth development programs related to self-esteem & other positive developmental measures. Youth participating primarily in sports & youth development programs had highest positive youth development scores	Positive Youth Development (competence, confidence, connection, character, caring)	77
[41] 2009	Quant.	Cross.	1,711	USA	10 to 18	Child. & youth	B	Compare activity patterns in sports & other types of organised activities for adolescents	Sport	Non sport organised extracurricular activities	Theory of Positive Youth Development	Those who participate in sports had more positive outcomes (including confidence, connections & social well-being) compared with those with little or no involvement in sport but less compared with those who participated in sport plus other activities	Positive youth development, social well-being, school connectedness,	86
[42] 2008	Quant.	Cross.	13,857	USA	12-18	Adol.	B	Examine the relative risk of hopelessness & suicidality associated with sport & physical activity participation	Team sport	No sport	-	Sport participation protected against hopelessness & suicide. Social support & integration may account for some of the differences between types of physical activity	Hopelessness, suicidality	80
[43] 2008	Quant.	Cross.	3836	USA	9th-12th grade	Adol.	B	Explore relationships between physical activity behaviours & emotional self-efficacy	Sport	No sport, other physical activity	-	Playing on sport teams was associated with better emotional self-efficacy	Emotional self-efficacy	80
[44] 2008	Quant.	Long.	201	Canada	8-11 & Parents	Child. & parents	B	Examine the role of organised sport participation as a moderator of the links between shyness & psychosocial maladjustment in childhood	Sport	no sport	-	Sport participation was positively related to social skills & self-esteem. Shy children who participated in sport reported a significant decrease in anxiety. Benefits of sport participation for children include higher positive affect & well-being & social skills	Assertive, self control, cooperation, self-esteem, positive affect, well-being	76
[45] 2008	Qual.	Cross.	55	USA		Adol.	B	Understand the positive and negative aspects of parental involvement in youth sports	Sport	-	-	Sport builds self-esteem, friendships and a sense of belonging among a team of peers (within a team or competing as an individual against peers).	Self-esteem, friendships, sense of belonging	NA
[46] 2006	Quant.	Cross.	449	Canada	8 th-10th grade	Adol.	B	Test hypothesis that positive team sports involvement mediates the effects of risks on depression	Team sport & positive team sport	Less or no team sport	-	Participation in team sports partially mediated the risks for depressive symptoms	Depressive symptoms	81
[47] 2006	Quant.	Cross.	203	USA	11-13	Child.	B	Examine relationship between children’s sport participation & emotional well-being	Sport	Less sport or no sport	-	Sports participation positively associated with self-concept. Greater participation in sports was related to enhanced emotional & behavioural well-being. Athletic competency was related to reduced emotional & behavioural problems	Self-concept, emotional & behavioural wellbeing, perceived competence	53
[48] 2006	Quant.	Cross.	382	Canada	5th-8th grade	Child. & Adol.	B	Examine the links between sports participation & self-esteem	Sport	Less or no sport	-	Sports participation was related to self-esteem. Physical self-esteem mediates the relationship between sports participation & general self-esteem	Competence, self-esteem	75
[49] 2006	Quant.	Cross.	7428	Switzerland	16-20	Adol.	B	Examines socio-demographic & lifestyle correlates of sport participation	Sport	No sport	-	Most active adolescents reported greater well-being than their inactive peers. Sport participants had higher perceived health & life satisfaction	Perceived health, life satisfaction	87
[50] 2004	Quant.	Long.	247	USA	M 13 Wave 1 & M 16 Wave 4	Adol.	F	Investigated the contribution of team sport to self-esteem development	Team sport achievement	-	-	Sports achievement experiences in early adolescence positively associated with self-esteem in middle adolescence	Self-esteem	67
[51] 2004	Quant.	Cross.	4758	USA	9th-12th grade	Adol.	B	Explore relationships between perceived life satisfaction & physical activity behaviour	Team sport	No team sport	-	Playing on team sports associated with greater life satisfaction	Life satisfaction	87
[52] 2003	Quant.	Cross.	51,168	USA	9th grade	Adol.	B	Investigate whether school team sports participation is associated with higher levels of psychosocial functioning & healthy behaviour than other activities	Team sport	Other extracurricular activities	-	Students involved in sport had higher self-image & less emotional distress than students not involved in sport	Emotional distress, suicidal behaviour	87
[53] 2003	Quant.	Cross.	450	USA	9th-12 grade	Adol.	B	Investigate different developmental & negative experiences in organised youth activities	Sport	Other organised activities	-	Youths in sport activities reported higher rates of managing emotions compared to youth in academic & leadership activities. Youth in sports reported higher rates of self-knowledge, emotional regulation & negative peer interaction	Self-knowledge, emotional regulation, peer interaction	88
[54] 2003	Quant.	Cross.	770	USA	M 16	Adol.	B	Compare the impact of organised, more intensive sports participation with lower intensity participation among high school student-athletes	Competitive sport participation	Recreation sport participation	-	Competitive sports participation associated with a lower frequency of mental ill-health	Mental health problems	60
[55] 2003	Quant.	Cross.	918	USA	16-17	Adol.	B	Examine the participation of adolescents in both constructive, organized & relaxed leisure activities	Sport	Other structured & unstructured activities	-	Youths highly involved in sports were more ‘psychologically resilient” or able to bounce back from problems	Psychological resilience	67
[56] 2002	Quant.	Cross.	4632	USA, Puerto Rica	M 15	Adol.	B	Test hypothesis that school-based sport is associated with self-esteem & school attachment & a sense of physical wellbeing mediates this relationship	School sport	Less or no school sport	-	Participating in school sport positively related to self-esteem	Self-esteem	67
[57] 2001	Quant.	Long.	500	Germany	12-18	Adol.	B	Investigate possible causal relationship between adolescent activity in sports club & improvements in motor ability & psycho-social health	Sports club member	Non-sports club member	Socialization theory & Ecology-oriented approaches	Sport club activities associated with positive psycho-social health (including self-esteem). Girls discover sports as a source of self-esteem earlier than boys	Self-esteem, social interactions	50
[58] 2001	Quant.	Long.	1036	USA	9th to 11th grade	Adol.	B	Investigate whether sports involvement positively contributes to mental health	Team sport	Less or no team sport	-	Team sport involvement associated with reduced depressed mood	Depressed mood	67
[59] 2001	Quant.	Long.	900	USA	6th-10th grade *initially*	Adol. & adult	B	Examined sequel of participation in high school activities & identity group	Team sport	Other extracurricular activities	-	Sport participation protects student athletes against social isolation	Social isolation	44
[60] 2000	Quant.	Cross.	89	USA	M 17	Adol.	B	Investigated whether sports involvement is related to social & psychological well-being	Sport	No or less sport	-	Moderate sports involvement group had lower depression scores than low sports involvement group	Depression score	60
[61] 2000	Quant.	Cross.	1769	USA	M 16	Adol.	B	Investigate effects of athletic participation in the development of adolescent mental health patterns	Team sport	No or less sport	-	Sport participation associated with mental health benefits	Mental health	73
[62] 1999	Quant.	Cross.	9268	Switzerl&	15-20	Adol.	B	Determine the direction & strength of the associations between frequency of sport & health variables	Sport & club sport	No sport	-	Sport participants had superior well-being (better adjusted, less nervous or anxious, more often full of energy & happy about their life, & less often sad, depressed or desperate, & less suicidal thoughts	Well-being, depressed, suicidal thoughts	80
[63] 1996	Quant.	Cross.	5076	UK	M 16	Adol.	B	Assessed association between extent of participation in regular sport or vigorous recreational activity & emotional wellbeing	Sport	Less or no sport	-	Sport & vigorous recreational activity participation was positively associated with emotional well-being	Emotional well-being	75
[64] 1993	Quant.	Long.	22	USA	Last 2 years of high school	Adol.	B	Examine the effects of participation in sport during last 2 years of high school	Sport participation	Non sport participation	Coleman's (1959) emphasizing the effects of sport participation for adolescents, & Synder’s (1985) multiple role theory	Sport participation positively associated with post-secondary outcomes of social self concept	Social self-concept	65

*Quant. (Quantitative): Qual. (Qualitative): Rev. (Review) ** Adol. (Adolescent): Child. (Children) ***M (Male): F (Female): B (Both Male and Female).

### Conceptual model development

**Figure 2:**
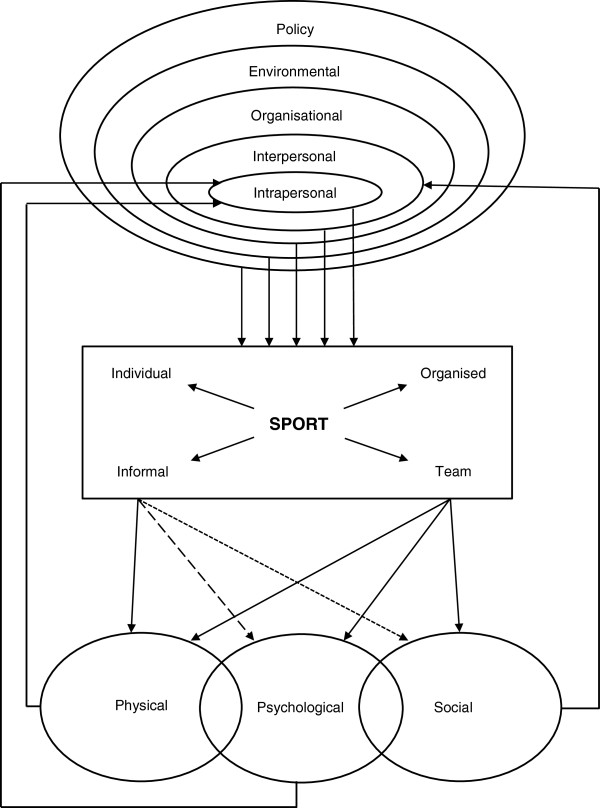
Health through Sport conceptual model.

Based upon the literature presented in this review, a conceptual model of Health through Sport has been developed (Figure 2). The model depicts the relationship between determinants driving sport participation and the reported psychological and social health benefits of participation. The terminology used in this conceptual model is as defined in the inclusion criterion 3 above. The determinants are represented as per the Socio-Ecological Model [19,65]. Upon reviewing the studies, two dimensions of sport participation were identified, and it became evident that some reported health benefits were more likely to be associated with some contexts of sport participation than others. Therefore, a model was developed to represent the two contextual dimensions of sport participation and the different strengths of association between different contexts of sport participation and the three health aspects (physical, psychological and social).

With regard to causality, we note that most studies have been cross-sectional and observational in nature, and hence do not provide strong evidence of causality. The literature suggests that sport can have positive health benefits; however it is also the case that better health may predispose people to initiate and maintain participation in sport. A few longitudinal studies provide stronger evidence of causality. However, in the absence of randomised and controlled experimental studies, which are challenging to implement in this domain, it will remain difficult to unequivocally determine the nature and direction of causality. Notwithstanding this, terms like ‘outcome’ and ‘benefit’ of sport participation have been used to describe the results of many of the studies reviewed, and we have used the same terminology in reviewing these studies.

## Results and discussion

A total of 3668 publications were initially identified. Table 1 provides a summary of the 30 studies that met the inclusion criteria. Since the studies were generally conducted within schools, they included school age children and adolescents, generally 18 years or less. Most studies were quantitative (n=26) rather than qualitative (n=3), with one study incorporating both quantitative and qualitative methods. There were no randomised controlled trials, and the majority of studies were cross-sectional and observational (n=21). Of the longitudinal studies (n=9), the time between data collection was generally between 1 and 3 years (n=7), with one study reporting 12 years between data collection periods. The sample sizes ranged considerably, from 22 participants to large national surveys of over 50,000 participants. The United States of America was the country where most studies were conducted (n=21), followed by Canada (n=4), Switzerland (n=3), and Germany, United Kingdom and Puerto Rica (n=1). One study was conducted with participants across two countries, the USA and Puerto Rica. The age ranges of the children and adolescents differed considerably across studies. Six studies incorporated data from both the child or adolescent and their parent(s).

Most studies scored highly on the modified Downs and Black scale of study quality (median 75 percent; range 33–88 percent). Those studies within the highest tertile score range were all cross-sectional quantitative studies [39,41-43,46,49,51-53,62]. Only one of the 10 studies in the highest tertile score range incorporated a theoretical approach - the Theory of Youth Development [41]. Half of these 10 studies investigated differences in health measures between participants in sport/club sport and either other organised activities or no sport [41,43,49,53,62]; the other half more specifically investigated team sport participation in comparison to less or no team sport [39,42,46,51,52]. There was no clear distinction between the key findings of higher and lower ranked studies; both high and lower quality studies reported similar associations between sport participation and the psychological and social health domains.

Prima facie, longitudinal studies can provide greater strength of evidence regarding causality than can cross-sectional research. However, all of the longitudinal studies reviewed [35,40,44,50,58] had other methodological limitations, and as a consequence were not represented in the highest tertile of study quality scores. The results of these studies were consistent with those of the cross-sectional studies.

There were few (n=2) qualitative studies, and similar health benefits of participation in sport were also reported in the quantitative studies. The study by Holt et al., (2011) provided more depth than was captured in the other studies reviewed. Interviews with parents and children unearthed a wide range of developmental benefits, both personal and social benefits [36]. Psychological aspects of emotional control and exploration were reportedly related to sport participation. In addition, social benefits of relationships with coaches and friends were reported in this study [36].

The investigation of health benefits through participation in physical activity mainly involved cross-sectional surveys conducted through schools. In most cases the students were not allocated to a participation group prior to the study, and as such there were no control groups. This limits the capacity to attribute causality of participation on health outcomes.

The psychological and social health measures in each study were diverse (Tables 1 and 2). The most common variables related to psychosocial functioning and emotional wellbeing (n=6), followed by risk of depression and mental ill health (n=5), developmental aspects/behaviour (n=4), social anxiety and shyness (n=3), self-esteem (n=3) and suicidal behaviour (n=3). Some studies (n=15) investigated the differences between sports and non-sports participants, but many did not distinguish between sport and other categories of PA. In the studies involving adolescents, it was common to investigate differences in youth behaviour and development according to their participation (or not) in out-of-school extracurricular activities. Sport was sometimes defined as ‘school sport’ , ‘club sport’ or ‘team sport’; however no studies investigated associations between specific types of sport and psychological or social health domains.

**Table 2:** Summary of the psychosocial health aspects associated with sport participation for children and/or adolescents

**Category**	**Specific health aspect**	**Study**
Psychological	**Assertive**	Findlay, 2008
Psychological	**Caring**	Zarrett, et al., 2009
Psychological	**Character**	Zarrett, et al., 2009
Psychological	**Competence**	Zarrett, et al., 2009; Donaldson, et al., 2006; Bowker, 2006
Psychological	**Confidence**	Zarrett, et al., 2009; Holt et al., 2011; Wiersma et al., 2008
Psychological	**Emotional control, exploration,**	Holt et al., 2011
Psychological	**Emotional regulation**	Hansen et al., 2003
Psychological	**Emotional self-efficacy**	Valois et al., 2008
Psychological	**Emotional wellbeing**	Donaldson, et al., 2006; Steptoe et al., 1996
Psychological	**Fewer depressive symptoms**	Boone et al., 2006; Gore et al., 2002; Sanders et al., 2000; Ferron et al., 1999
Psychological	**General health perceptions**	Snyder et al., 2010
Psychological	**Less emotional distress**	Harrison et al., 2003
Psychological	**Less hopelessnes**	Talliaferro, 2008
Psychological	**Less suicidality**	Taliaferro et al., 2011; Talliaferro, 2008; Harrison, et al., 2003; Ferron et al., 1999
Psychological	**Life satisfaction**	Michaed et al., 2006; Valois et al., 2004
Psychological	**Mental health**	Snyder et al., 2010; Pyle, et al., 2003
Psychological	**Positive affect**	Findlay, 2008
Psychological	**Psychological resilience**	Bartko et al., 2003
Psychological	**Self control**	Findlay, 2008
Psychological	**Self-concept**	Donaldson, et al., 2006;
Psychological	**Self-esteem**	Pedersen, et al., 2004; Erkut et al., 2002; Brettschneider, 2001; Wiersma et al., 2008; Findlay, 2008; Bowker, 2006
Psychological	**Self-knowledge**	Hansen et al., 2003
Psychological	**Try to resolve conflicts**	Howie et al., 2010
Psychological	**Wellbeing**	Findlay, 2008, Ferron et al., 1999
Psychosocial	**Behavioural wellbeing**	Donaldson et al., 2006
Psychosocial	**Connectedness**	Linver et al., 2009; Zarrett, et al., 2009
Psychosocial	**Perceived health**	Michaud et al., 2006
Psychosocial	**Reduced social anxiety**	Dimech et al., 2011
Psychosocial	**Youth development**	Linver et al., 2009
Social	**Cooperation**	Findlay, 2008
Social	**Relationships with coaches, friends**	Holt et al., 2011
Social	**Show respect for teachers and neighbours**	Howie et al., 2010
Social	**Social functioning**	Snyder et al., 2010
Social	**Social interaction/integration; Social skills**	Hansen, et al., 2003; Brettschneider, 2001; Wiersma et al., 2008; Howie et al., 2010; Holt et al., 2011
Social	**Social self-concept**	Marsh, 1993
Social	**Social well-being**	Linver et al., 2009
Social	**Sportsmanship**	Wiersma et al., 2008
Social	**Teamwork**	Wiersma et al., 2008

Table 2 provides a broad overview of the health outcomes found to be significantly and positively associated with sports participation, and lists the studies that reported each health outcome. The most common positive outcomes were higher self-esteem (n=6 studies), better social skills (n=5 studies), fewer depressive symptoms (n=4 studies), higher confidence (n=3 studies) and higher competence (n=3 studies) amongst sport participants than non-sport participants. In total 40 different psychological and social health factors were reportedly associated with participation in sport.

In general, there were few theoretical constructs used to frame or explain the research findings. Only six studies (20%) incorporated theoretical or conceptual constructs. The most frequently adopted construct (n=3) was the theory of Positive Youth Development [36,40,41], which propounds the notion that children are ‘resources to be developed’ rather than ‘problems to be solved’, and that all youth have the potential for positive development [66].

One study that incorporated the theory of Positive Youth Development [36] also utilised an ecological approach, whereby the study was exploratory and not guided by one specific theory. In this case these researchers investigated the intrapersonal and interpersonal benefits of participation in sport. Similarly, an ecological approach has been combined with other theories such as the Socialisation Theory [57]. Brettschneider (2001) proposed that there are many contributing factors to the relationship between sports club participation and adolescent development [57]. As such, a multivariate structure, as well as cumulative and interactive effects, needs to be taken into account. Secondly, within his theoretical framework Brettschneider proposes that each individual is assumed to be the creator of his/her development. Whilst studies often discussed theories underpinning the research, it was not always clear how particular theories were incorporated into the methodology. For example Holt et al., introduced the Positive Youth Development theory in their introduction, but there was no mention of how this was applied in the methodology of data collection or in the analysis and interpretation [36]. On the other hand, Zarrett et al. clearly defined how they measured and indexed Positive Youth Development [40].

A recent study [37] incorporated Antonovsky’s Salutogenesis model [67] and Bandura’s theory of Social Learning [68]. The foundation of Antonovsky’s model is that heterostasis, ageing and progressive entropy are core characteristics of all living organisms. The model focuses on what makes a person maintain good health rather than focusing on the aetiology of sickness. In terms of the Social Learning theory, it is suggested that organised sport, particularly in teams, could be an important factor in a child’s social development [37]. However, this was a general discussion comment, and it is not clear how the Social Learning Theory was applied in the methodology of this study [43].

The theoretical perspective of Marsh [64] was adopted from Coleman’s [69] seminal work which “implies a zero-sum model in which greater involvement in extracurricular activities necessitates a decreased involvement in more narrowly defined academic pursuits” (p.19) in a way that is complementary rather than multiple roles being in conflict [64]. Stemming from Coleman’s earlier work, Marsh discussed Snyder et al. (1995) Multiple Role theory [70] which proposes that adolescents take on multiple roles as both a student and an athlete. Marsh suggests that “multiple roles may create psychological stress based in part on time and energy limitations, multiple roles may be complementary and may lead to energy expansion” (p19). In essence Marsh attempts to capsulate the perspective that sport participation as an additional extracurricular activity can have positive outcomes, rather than sport being seen, as depicted in earlier theoretical perspectives, as a burden, taking time away from academic pursuits. However, as with a number of other studies reviewed, it was not clear how the particular behavioural theory was applied in the study [64].

Few differences were evident between the conclusions of studies of higher and lower quality or of different study design. There were however, clear differences in the reported health outcomes associated with different contexts of participation. Therefore the following presents and discusses the reported psychological and social health benefits of participation in sport in the different contexts of: extracurricular activities; team sport; school or club sport; and sport in general. These categories, which are not mutually exclusive, were based upon the definitions or categorisation made within each individual study. Furthermore, the health benefits according to different types of participation are discussed. Lastly, given the greater strength of evidence regarding causality in longitudinal versus cross-sectional research, the key findings from the longitudinal studies are summarised.

### Extracurricular activities

Several studies have investigated the influence of sport, as one type of extracurricular activity, on positive youth development [36,40,41] general behaviours [39] and personal development [53]. Other extracurricular activity categories considered were school-based activities, religious activities, youth groups, performing arts, volunteering, paid work, band and music lessons [40,41,52]. The definition of ‘sport’ as an extracurricular activity varied considerably. Sport was sometimes defined as including both team and individual sports [40,53] or encompassing different categorical groups for both team and individual sports participants [37], whilst others categorised groups as structured versus unstructured activities [55]. Howie et al. (2010) investigated extracurricular (outside school) activities - sports teams/lessons, sports clubs/organisations, or both - in the previous year [39].

While the qualitative study of Holt et al. (2011) did not compare sports participation with other activities, parents reported benefits for their children in personal and social development from sport participation. Social benefits included positive relationships with coaches, making new friends, and developing teamwork and social skills. Personal benefits included children being emotionally controlled, enjoying exploration, having confidence and discipline, performing well academically, managing their weight and being ‘kept busy’ [36].

Similarly, Bartko and Eccles (2003) reported that structured activities (sport being one of them) led to higher positive functioning for participants [55]. Howie et al. (2010) reported that children participating in both sports and clubs had higher social skill scores compared with children who did not participate in any outside-school activity [39]. Concurring with these findings, Linver et al. (2009) found that participation in sport and other organised activities had the greatest youth development outcomes, and low involvement in organised activities outside school was associated with less positive development across the board [41]. Sports participation alone had more developmental benefits than non-participation or other types of extracurricular activities, however the greatest benefits were seen for those involved with both sport and other activities [39,41].

Whilst positive social aspects of participation in sport have been consistently reported, it has also been found that young people involved with sport had higher rates of negative peer interaction [53]. These researchers concluded that this may be due to the competitive nature of sports activities compared to other activities. Even so, they found that, in addition to physical benefits, those involved with sport had higher rates of self-knowledge and emotional regulation than those involved with other activities [53]. While Harrison et al. (2003) defined team sport separately from other activities, their results were collated as sports only, activities only and sports and activities [52]. Contrary to some other findings, they found that sports alone (and also in combination with other activities) were associated with significantly better health outcomes, including higher healthy self-image and lower risk of emotional distress, suicidal behaviour and substance abuse.

Two longitudinal studies, one with a year between measurements and another three years, investigated the effects of participation in extracurricular activity on youth development [40] and social anxiety [37]. Dimech and Seiler (2011) investigated sport only, categorised as non-participation, individual or team involvement [37], whereas Zarrett et al. (2009) investigated team or individual sport participation in comparison to participation in development programs, performing arts, arts and crafts, school clubs, volunteering, religious groups, and paid work [40]. Consistent with the cross-sectional results of Linver et al. (2009) and Howie et al. (2010), Zarrett et al. (2009) concluded that a combination of sport plus other youth development programs was related to positive youth development, even after controlling for total time spent in the activities and the duration of sport participation.

Dimech and Seiler (2011) measured the effects of extracurricular participation in sport on social anxiety [37]. Comparing team sport, individual sport and no sport, they reported an interaction between sport mode and time, with team sport participants having reductions in social anxiety scores over time, whilst anxiety scores in the no-sport and individual-sport groups actually increased. Dimech and Seiler concluded that sport practice had a positive effect as a buffer against anxiety, but only team sport and not individual sport.

### Team sport

Whilst some studies highlighted the benefits of extracurricular sport, the focus was more commonly on ‘team sport’ in general, without distinguishing between in-school and out-of-school settings [42,43,46,50,51,58,59,61].

The psychological and social health aspects measured included mental health benefits [61], social isolation [59], depressed mood and symptoms of depression [46,58], self-esteem [50], life satisfaction [51], hopelessness and suicidality [42] and emotional self-efficacy [43].

Cross-sectional studies included a survey of US high school students, in which participation in team sport was associated with lower general risk-taking and fewer mental health and general health problems compared with non-participation [61]. In another cross-sectional survey, team sport involvement was positively associated with social acceptance and negatively associated with depressive symptoms [46]. Boone and Leadbeater concluded that benefits from team sport may be related to the effect of positive experiences (in coaching, skill development, peer support) in enhancing perceived social acceptance and reducing body dissatisfaction [46]. Team sport participation has also been reported to protect against feelings of hopelessness and suicidality, even after controlling for levels of physical activity [42]. Another reported health benefit of participation in team sport (both school and extracurricular participation) is life satisfaction [51]. A study investigated the relationship between different physical activity behaviours, distinguishing between vigorous and moderate levels as well as strength/toning and team participation contexts, and found that meeting recommended levels of PA and participation in sports teams was significantly associated with better emotional self-efficacy [43].

In a longitudinal study of adolescents with measurements one year apart, team sport participation was found to be protective against depressed mood associated with school performance levels [58]. In a longitudinal study of females, team sport achievement experiences in early adolescence were positively associated with self-esteem three years later in middle adolescence [50]. Another longitudinal study spanning 12 years found that participation in team sport (specifically school teams) was associated with lower social isolation later in life, compared with other activities categorised as pro-social, arts, and school-based [59].

### School and/or club sport

Some studies distinguished between participation in ‘school sport’ and ‘club sport’ [38,54,56,57,62]. Snyder et al. (2010) while reporting school and club participation, then combined them into a single ‘athletes’ category and compared them to non-athletes on health-related quality of life measures. The athletes reported higher scores on physical functioning, general health, social functioning and mental health scales and a mental composite score, and lower on a bodily pain scale, than non-athletes [38]. Similarly, in a Swiss study, Ferron and colleagues classified adolescents as ‘athletes’ or ‘non-athletic’ on the basis of sports club participation. The athletes had superior well-being, including being better adjusted, feeling less nervous or anxious, being more often full or energy and happy about their life, feeling sad or depressed less often and having higher body image and fewer suicide attempts [62].

One longitudinal study of club sport participation over a three year period during adolescence in Germany, as well as identifying physical benefits, showed that sport club activities had a positive influence on the development of self-esteem, with girls discovering sports as a source of self-esteem earlier than boys [57]. In terms of relationships with peers and parents, club sport members did not differ significantly from non-members. Brettschneider and colleagues concluded that although sports club participants had better health outcomes, these benefits were due to self-selection bias rather than a sport club effect [57]. These researchers also acknowledged that research into the impact of sports by discipline, and studies of longer duration, are required.

In relation to school sport specifically, participation was found to be significantly associated with self-esteem in Latino subgroups of students living in the United States of America [56]. This was true for Mexican girls and boys, Puerto Rican girls and Cuban boys but not Puerto Rican boys and Cuban girls. Pyle and colleagues investigated participation in school sports defined as being high or low intensity. Participation in competitive sports was found to be associated with lower frequency of mental health problems [54].

### Level of sport involvement

Most studies defined sport participation as a binary categorical variable without further information regarding level of involvement. However, a few studies have investigated psychological and social health outcomes in relation to different levels of intensity of sport activities (low, moderate, vigorous, or high) [60,63] or frequency of participation and number of sport activities [48].

Steptoe and Butler (1996) assessed the association between extent of participation in sport or vigorous recreational PA and emotional wellbeing in adolescents [63]. Without distinguishing between sport and other vigorous PA, Steptoe and Butler reported that greater participation in vigorous activities was associated with lower risk of emotional distress [63]. Sanders and colleagues found that for high school senior students moderate sport participation (3–6 hours per week) was associated with lower depression scores than low sport involvement (0–2 hours) [60]. Donaldson and Ronan (2006) investigated participation in both “formal” and “informal” sports and reported that greater participation was related to enhanced emotional and behavioural well-being. Those participating in more formal sports reported significantly lower levels of emotional and social problems compared to those participating in fewer formal sports [47]. Another study investigated frequency of extracurricular sport and perceived health, health attitudes and behaviour [49]. Those with greater frequency of participation (at least twice per week) had better feelings of well-being compared to those who participated less than once per week [49]. One study looked at number of sports, type of sport, and years participating in sport, and found that sport participation was positively related to self-assessments of physical appearance and physical competence, physical self-esteem and general self-esteem [48]. Furthermore, these researchers found that differences between competitive and non-competitive sports was minimal, and suggested that for young adolescents, it is more important to consider the total number of sports and total number of years in sports-related activities [48].

### Sport in general

A few studies used a broad definition of sport without providing further context of participation [35,44,64]. Sport participation versus no sport participation was found to be significantly associated with enhanced self-concept [64]. A longitudinal study also reported benefits of participation in sport compared to no participation, in relation to lower rates of suicidal ideation including both thoughts and intentions [35]. In terms of the effect of sport participation on shyness, a longitudinal study with measurement at baseline and one year later found that sport was positively associated with positive adjustment (e.g. social skills and self-esteem) and that sport played a uniquely protective role for shy children, with shy children who participated in sport over time reporting significant decreases in anxiety [44]. Similarly, in a qualitative study of focus groups of parents of young people participating in sport, social factors as well as life skills and self-concept were stated as benefits of participation [45].

### Longitudinal studies

Longitudinal studies can provide stronger evidence of causality than cross-sectional studies. However, the longitudinal studies reviewed were generally short in duration, usually with only two measurement points, one or two years apart [35,40,44,50,58]. They were all observational in nature, with no control groups, and with limited measurement of the level of participation and frequency or duration of sport activities. All studies were based on surveys conducted through schools, with participation in sport and other extracurricular activities reported mainly in binary categories.

The main findings were that, after controlling for factors such as income, parents’ education, age and ethnicity, compared to no participation or participation in individual sports, participation in team sport had resulted in benefits such as lower social anxiety [37], lower social isolation [59], better social self-concept [64], and improved self-esteem [50]. Sport in general has also been associated with positive youth development [40]; the young people who were highly engaged in general, and those who participated primarily in sports and youth development programs, had the highest positive youth development scores.

In a recent study undertaken longitudinally over a one-year period, where sport participation was generally reported to be of 1–2 hour duration per week, there was no effect of weekly hours of sport on social anxiety [37]. Similarly, Findlay and Coplan (2008) in a longitudinal analysis over a one-year period, did not find significant effects of sport participation over the year (neither main effects of time or participation-time interactions) on social skills, self-esteem, positive adjustment or externalising problem behaviours [44]. However, shy children who participated in sport over a one-year period demonstrated a decrease in anxiety over time. Sport was associated with positive psychological and social outcomes, including higher positive affect and well-being and greater social skills. Shy and aggressive children who participated in sport reported higher self-esteem [44]. A study of club sport members compared to non-club members also did not show a systematic effect of club membership on most measures of psychological and social health in adolescents over three years [57]. Notwithstanding, clubs had a positive effect on adolescent self-esteem and were reported, on the basis of high membership rates, to be a highly integrative social force [57].

A US study in which high school students were interviewed at two time points one year apart, showed that for females, but not for males, team sport involvement was protective against depressed mood state associated with poor school performance [58]. Another US study of female adolescents over three years found that sports achievement experiences in early adolescence were positively associated with self-esteem in middle adolescence [50]. Team sports achievements, team sports self-evaluations and individual sports self-evaluations tended to be significantly and positively associated both cross-sectionally and longitudinally. Team sport achievement in early adolescence was related to girls’ global self-esteem in middle adolescence, and team sport self-evaluations mediated the relation between achievement and self-esteem. In addition, the relationship between achievement and self-esteem was partially mediated by girls’ perceptions of competence and interest in team sport, and mastery in team sport contributed to global self-esteem development [50].

Another longitudinal study showed that adolescents involved with team sport had lower suicide ideation with regard to both contemplation and intention [35]. These researchers suggested that when young people discontinue playing sport they lose the protective social networks, as well as connections to caring adults and pro-social peers, that help to promote healthy youth development and reduce the risk of suicide.

### Conceptual model

A conceptual model of Health through Sport is proposed that is based on three primary categories of outcome: physical, psychological and social, and two secondary categories: physical/psychological – aspects involving both the physical and psychological elements, and psychosocial – aspects involving both psychological and social elements.

While our model incorporates all five categories and thus depicts the full range of health aspects, the ‘physical’ aspects have been well reviewed elsewhere [1], and so this paper in focused on the psychological and social aspects, as defined above. Furthermore, while the present review was limited to research into children and adolescents, the general form of the Health through Sport model is believed to also apply to adults, although it is likely there would be some change in the specific elements of each component.

The model includes three major elements: determinants of sports participation, sport itself, and health outcomes of sport participation. The ‘determinants’ element is based on the well-established social ecological model [19,65] and is represented as concentric rings spreading out from the individual’s intrapersonal characteristics to widening spheres of influence. The sport element incorporates two dimensions of context: individual – team, and informal – organised, each of which is almost dichotomous, but also has some intermediate variants (e.g. running alone, running in an informal group, running for a club team, running in a club relay team). The three types of health outcomes - physical, psychological and social, are shown as overlapping, representing the fact that there may be interactions and interrelationships between physical and psychological aspects and between psychological and social health aspects. For example, there are relationships between physical fitness and mental state; and interpersonal relationships may satisfy needs for belongingness and, as such, influence psychological health. Another example is resilience, whereby psychological health may influence an individual’s capacity to engage in interpersonal relationships.

The different strengths of the various linkages between the sport element and the health outcomes represent the notion that all forms of sport contribute strongly to physical health, but that while organised and/or team forms also contribute strongly to psychological and social outcomes, informal and/or individual forms contribute somewhat less to psychological outcomes and relatively little to social outcomes. Finally, we have noted the limited evidence of causality in the literature reviewed. This ambiguity or reciprocity could perhaps be represented by double-headed arrows linking the physical, psychological and social elements to the sport element, but we have represented it by ‘feedback loops’ from the three outputs to the intrapersonal and interpersonal determinants.

### Limitations

This systematic review has some limitations. Whilst the search strategy, based on a-priori inclusion and exclusion criteria, was comprehensive and encompassed grey literature which reported primary data, conference proceedings were not included. Nor were non-English language articles included. The studies reviewed included a wide range of aims, focuses, measurement tools and indicators of both sport participation and health outcomes. This diversity of focus and methodology limited the extent of synthesis and precluded meta-analysis. Most studies were cross-sectional and used self-report measures. Therefore results should be interpreted with caution, and any conclusions regarding causation are conjectural.

## Conclusion

There is substantive evidence of many different psychological and social health benefits of participation in sport by children and adolescents. Furthermore, there is a general consensus that participation in sport for children and adolescence is associated with improved psychological and social health, above and beyond other forms of leisure-time PA. More specifically, there are reports that participation in team sports rather than individual activities is associated with better health. It is conjectured that this is due to the social nature of team sport, and that the health benefits are enhanced through positive involvement of peers and adults. However, the research is predominantly based on cross-sectional studies.

In light of the research evidence, acknowledging that research to date is predominantly based on cross-sectional studies, it is recommended that community sport participation is advocated as a form of leisure time PA for children and adolescents; in an effort to not only improve the obesity crisis associated with low PA levels, but to enhance other psychological and social health outcomes. It is also recommended that the causal link between participation in sport and health be further investigated and the conceptual model of health through sport tested. Furthermore, in light of the fact that our assessment of the quality of the studies to date has revealed considerable variation in study quality, it is recommended that researchers should give more attention to protocols such as CONSORT [71] and STROBE [72] in order to ensure high levels of methodological rigor in future studies.

## Abbreviations

PA: Physical activity.

## Competing interests

The authors declare that they have no competing interests.

## Authors’ contributions

RME contributed to the study design, the review of literature, analysis of literature, model conceptualisation, manuscript conceptualisation and preparation. JAY contributed to the study design, the review of literature, analysis of literature, model conceptualisation, manuscript conceptualisation and preparation. JTH contributed to analysis of literature, model conceptualisation and representation, and manuscript preparation. MJC contributed to analysis of study quality and critical review of the manuscript. WRP contributed to the study design and critical review of the manuscript. All authors read and approved the final manuscript.
